# Modulation of Signal Strength Switches Notch from an Inducer of T Cells to an Inducer of ILC2

**DOI:** 10.3389/fimmu.2013.00334

**Published:** 2013-10-22

**Authors:** Rebecca Gentek, J. Marius Munneke, Christina Helbig, Bianca Blom, Mette D. Hazenberg, Hergen Spits, Derk Amsen

**Affiliations:** ^1^Department of Cell Biology and Histology, Academic Medical Center, Amsterdam, Netherlands; ^2^Department of Hematology, Academic Medical Center, Amsterdam, Netherlands; ^3^Tytgat Institute for Liver and Intestinal Research, Academic Medical Center, Amsterdam, Netherlands; ^4^Sanquin Research and Landsteiner Laboratory, Department of Hematopoiesis, Amsterdam, Netherlands

**Keywords:** innate lymphoid cells, ILC2, Notch, human, T cells, signal strength, thymus

## Abstract

Innate lymphoid cells (ILCs) are emerging key players of the immune system with close lineage relationship to T cells. ILC2 play an important role in protective immunity against multicellular parasites, but are also involved in the pathogenesis of type 2 immune diseases. Here, we have studied the developmental requirements for human ILC2. We report that ILC2 are present in the thymus of young human donors, possibly reflecting local differentiation. Furthermore, we show that uncommitted lineage^−^CD34^+^CD1a^−^human thymic progenitors have the capacity to develop into ILC2 *in vitro* under the influence of Notch signaling, either by stimulation with the Notch ligand Delta like 1 (Dll1) or by expression of the active intracellular domain of NOTCH1 (NICD1). The capacity of NICD1 to mobilize the ILC2 differentiation program was sufficiently potent to override commitment to the T cell lineage in CD34^+^CD1a^+^ progenitors and force them into the ILC2 lineage. As Notch is an important factor also for T cell development, these results raise the question how one and the same signaling pathway can elicit such distinct developmental outcomes from the same precursors. We provide evidence that Notch signal strength is a critical determinant in this decision: by tuning signal amplitude, Notch can be converted from a T cell inducer (low signal strength) to an ILC2 inducer (high signal strength). Thus, this study enhances our understanding of human ILC2 development and identifies a mechanism determining specificity of Notch signal output during T cell and ILC2 differentiation.

## Introduction

A new group of hematopoietic effector cells has been identified in recent years, which is now commonly referred to as the innate lymphoid cell (ILC) family (1). Cells belonging to this family characteristically express the IL7Rα chain (CD127), but lack markers specific for any other hematopoietic lineage. Members of this family are closely related to T cells, but do not express rearranged antigen receptors (2).

The ILC family displays broad functional diversity, which strikingly resembles that of T cells in terms of activating stimuli and signature cytokines secreted. For every T helper cell lineage, there appears to be a corresponding ILC lineage: group 1 ILC produce IFNγ like T helper 1 cells (Th1) (3–5), ILC2 produce Interleukin 5 (IL-5) and IL-13 and are thus akin to Th2 cells (6–9), and members of the ILC3 lineage produce IL-17 and/or IL-22, resembling Th17 and Th22 cells (10–12). ILC secrete these factors in response to cytokines produced by epithelial and myeloid cells upon stress inflicted by pathogens. For instance, type 2 ILC respond to IL-25, IL-33, and thymic stromal lymphopoetin (TSLP), which are secreted by mast cells and epithelial cells (13). ILC2 are crucial for defense against helminth parasites (6) and involved in airway inflammation and tissue repair associated with influenza infections (14, 15). Accumulating evidence also implicates ILC2 in the pathogenesis of type 2 inflammatory diseases such as allergic asthma (16–20).

Like T cells, ILC2 are known to derive from a common lymphoid progenitor (CLP) (21–23). While developmental processes of ILC are only beginning to be explored, differentiation of T cells has been well characterized. T cell differentiation is initiated after migration of CLP to the thymus, the primary site of T cell development. Developing human T cells can be separated into several discrete stages. The earliest thymic progenitors express CD34, but lack expression of CD7 and CD1a (24, 25). These cells have been shown to retain myeloid potential *in vitro* (26), whereas CD7 upregulation restricts them to NK/T potential. Commitment to the T cell lineage is marked by upregulation of CD1a (25). This is followed by rearrangement of T cell receptor β genes. Once a fully rearranged in frame TCRβ gene is generated, its gene product combines with the pre-TCRα chain (pTα) to form the pre-TCR, allowing a process called β-selection to take place. In humans, TCRβ^+^ cells first appear at an immature CD4^+^ stage (ISP4^+^) stage (27). As a consequence of β-selection, cells expand massively, (further) upregulate CD4 and CD8 co-receptors and rearrange their TCRα genes to generate the mature TCR, which is subjected to positive and negative selection processes. Final differentiation of T cells into effector cells, such as Th1, Th2, or Th17 cells, does not occur until the cells are activated by cognate antigen in the secondary lymphoid organs.

Aside from the absence of antigen receptors, ILC clearly are distinct from T cells in their developmental requirements. Thus, ILC lineages depend on Id2 for their development, whereas this factor is dispensable for T cell development. Also, the factor RORα is essential for differentiation of ILC2 cells, but is not required for development of the corresponding Th2 subset, at least *in vitro* (20). Nonetheless, many parallels do exist between the factors that regulate differentiation of the various Th subsets and their ILC counterparts. For instance, RORγt is required for generation of (murine) Th17 and group 3 ILCs (28), whereas evidence suggests that the lineage defining transcription factor for Th1 cells, Tbet (29), also regulates ILC1 differentiation (30). ILC2, on the other hand, depend on GATA3 for development and function, as do Th2 cells (31–34). Two additional factors known to govern T cell specification from thymic progenitors were recently shown to also be required for ILC2 differentiation, namely Tcf1 (35) and Notch (23).

Notch is a cell surface receptor, which is activated by binding to membrane bound ligands of the Delta like (Dll1 and Dll4) and Jagged (Jagged 1, Jagged 2) families. Ligand binding initiates a proteolytic cascade, which results in the release of the intracellular portion of the receptor, the Notch intracellular domain (NICD). NICD then translocates to the nucleus, where it associates with the DNA binding factor CSL [named after CBF-1 (mammals), Su(H) (*Drosophila*), and Lag-1 (*C. elegans*)]. Together, NICD and CSL recruit additional coactivators to induce transcription of target genes (36).

Notch signaling is absolutely required for at least two steps during the differentiation of T cells: T cell commitment and β-selection. The Notch pathway has also been implicated in differentiation of ILC (37). Lymphoid tissue inducer cells (LTi), which belong to the ILC3 group (1), require transient Notch activity at an early stage but Notch needs to be downregulated to allow for further differentiation of these cells (38). Depending on the microenvironment, Natural Cytotoxicity Receptor (NCR)^+^ ILC3 (named according to the expression of the NCR genes Nkp44/Nkp46) also might develop under the influence of Notch (39, 40). Finally, murine studies have demonstrated that group 2 ILC require Notch signaling for their development *in vitro* (23, 35). Whether Notch also regulates differentiation of human ILC2 has not been examined.

The involvement of Notch in differentiation of both ILC2 and T cells raises the question how activation of these pathways results in adoption of the T cell versus the ILC2 differentiation program. Two fundamentally different mechanisms are possible. First, the two cell types develop from different precursors, already more or less committed to either lineage. Alternatively, a common precursor gives rise to both cell types. In this scenario, the signals driving differentiation are distinct either qualitatively, involving additional signals dedicated to either lineage, or quantitatively.

Here, we examined these possibilities by studying Notch mediated *in vitro* differentiation of human thymocytes. We find that human thymic progenitors can give rise to both T cells and ILC2 in response to activation of Notch. Our data show that the strength of the Notch signal determines whether T cells or ILC2 are generated, with stronger signals favoring ILC2 differentiation. Thereby, we provide a mechanism explaining how the distinction between the T cell and ILC2 program can be made.

## Results

### Group 2 innate lymphoid cells can be found in the human thymus

**Figure 1:**
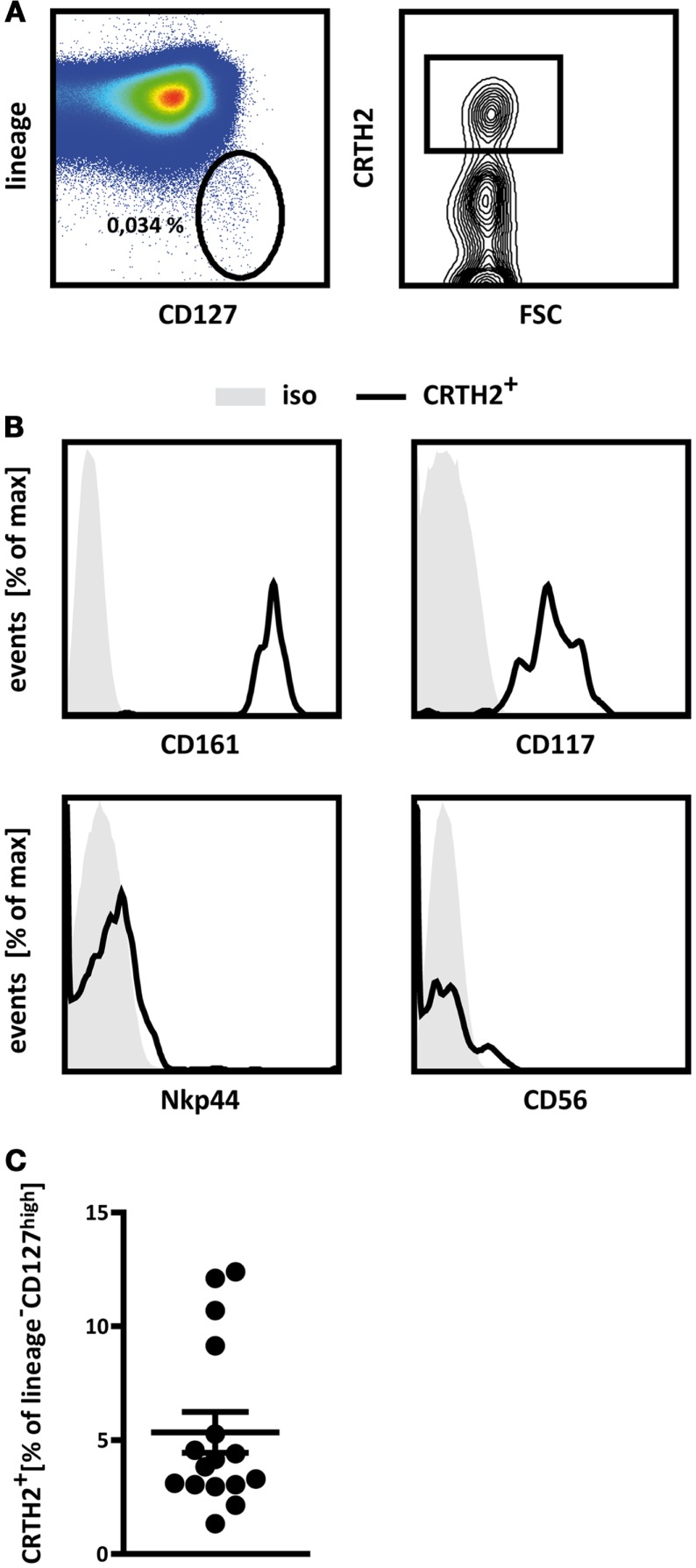
**Human thymus contains group 2 innate lymphoid cells**. **(A)** Gating strategy applied to identify thymic ILC2. Thymocytes were pre-gated for CD45^+^ cells and innate lymphoid cells were defined as lineage^−^ (CD1a^−^CD3^−^CD11c^−^CD14^−^CD19^−^CD34^−^CD123^−^TCRαβ^−^TCRγδ^−^BDCA2^−^FcϵRI^−^) CD127^high^ cells (left). This population was further analyzed for the presence of CRTH2^+^ cells (right). Percentages of cells in the respective gates are displayed. **(B)** Flow cytometric analysis of the expression of CD161, CD117 (cKit), Nkp44, and CD56 by thymic ILC2 as defined in **(A)**. Representative FACS plots from a single donor are shown **(A,B)**. **(C)** Relative abundance of ILC2 in human thymic specimens from a total of 16 donors analyzed **(C)**. Results in **(C)** are shown as mean ± SEM.

The close lineage relationship between T cells and group 2 ILCs (35) suggests that common precursors may exist for both lineages, which, depending on microenvironmental signals, differentiate into either T cells or ILC2. T cells differentiate in the thymus. Where ILC2 develop has not been established. There is evidence that these cells are generated in other locations than thymus, as suggested by the discovery of an ILC2 precursor in bone marrow (32, 41) and the presence of ILC2 in athymic FoxN1^nu/nu^ (nude) mice (23, 42). However, these findings do not exclude the possibility that ILC2 can be generated in the thymus as well. If common precursors of ILC2 and T cells exist, which differentiate into ILC2 in the thymus, one would expect to find differentiated ILC2 in this organ. To examine this, we analyzed single cell suspensions of freshly isolated human thymocytes by multi-color flow cytometry for markers associated with group 2 ILC. ILCs were defined as CD45^+^ cells expressing high levels of the IL7Rα chain (CD127), but lacking expression of markers specific for T cells (CD1a, CD3, TCRγδ, TCRαβ), B cells (CD19), hematopoietic stem cells (CD34), and other lineages (CD11c, CD14, CD94, CD123, FcϵR1, BDCA2), collectively termed “lineage” (Figure 1A, left). We found that an average of 0.0625% of CD45^+^ thymocytes belong to the ILC lineages. We further analyzed these IL7Rα^high^lineage^−^cells for the expression of CRTH2 (Figure 1A, right), which, within the family of ILCs, is specific for group 2 ILC (9). Using this strategy, we indeed detected a distinct population of CRTH2^+^ ILC in thymic specimens, on average accounting for 5% of the lineage^−^IL7Rα^high^ ILC compartment (Figure 1C).

Phenotypically, thymic lineage^−^IL7Rα^high^CRTH2^+^ cells are similar to group 2 ILCs identified in other human tissues in that they express CD161 (9) and cKit (CD117) (Figure 1B), as was described for a subpopulation of human ILC2 (1). Furthermore, thymic lineage^−^IL7Rα^high^CRTH2^+^ cells do not express CD56 or NKp44 (Figure 1B), which are associated with conventional NK cells and ILC3 (1). These cells therefore adhere to what have been defined as the phenotypic hallmarks of human group 2 ILC (2, 9) and can thus be regarded as thymic ILC2.

The presence of ILC2 in the thymus is remarkable, because thus far, such cells have only been found in mucosal tissues in the intestine and airways, consistent with their role in innate border patrol, in blood (9), presumably reflecting their migration, and in bone marrow, the major site of hematopoiesis (8, 34). Although we did not formally test whether ILC2 differentiation takes place in the human thymus, the fact that such cells are found at relatively high numbers in the thymus is consistent with the possibility that these cells are generated in this non-inflamed organ.

### *In vitro* differentiation of ILC2 from thymic progenitors by notch signaling

**Figure 2:**
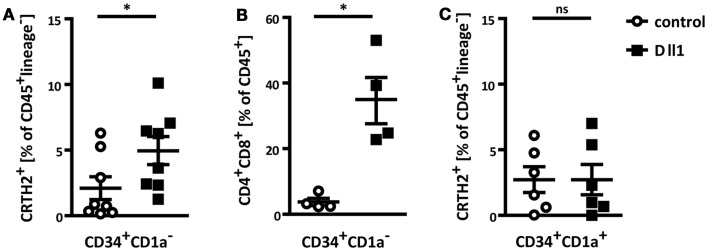
**Thymic progenitors can differentiate into ILC2 *in vitro* by Notch activation**. **(A)** Flow cytometric analysis of ILC2 differentiation from thymic CD34^+^CD1a^−^ progenitors. Cells were cultured on control OP9 or OP9 Dll1 as indicated for 1 week. ILC2 were defined as CD45^+^lineage^−^CRTH2^+^. **(B)** CD34^+^CD1a^−^ progenitors were cultured on OP9 cells as indicated. After 2 weeks, the abundance of CD4^+^CD8^+^ T cells was assessed. **(C)** Thymic CD34^+^CD1a^+^ progenitors were cultured and ILC2 development was measured as in **(A)**. Data are shown as mean ± SEM from minimally four independent experiments. Mann–Whitney test was performed for statistical analyses, *p < 0.05.

Given the presence of innate group 2 lymphocytes in the human thymus, we asked whether these cells can develop directly from thymic progenitors. A recent study showed that ILC2 differentiation can be induced from murine bone marrow-derived common lymphoid and thymic progenitors by co-culture with OP9 stromal cells expressing the Notch ligand Dll1 (23). To test whether human thymic progenitors have the capacity to differentiate into ILC2, we initiated OP9 co-cultures with human CD34^+^CD1a^−^ thymocytes, which have not yet committed to the T lineage (43). Although some lineage^−^IL7Rα^+^CRTH2^+^ cells appeared after 1 week of culture with control OP9 cells, the frequency of such cells was significantly increased upon co-culture with OP9 Dll1 (Figure 2A). Under these same conditions, OP9 Dll1 cells also induced differentiation of T cells, as expected, but this process required minimally 2 weeks of co-culture (Figure 2B).

Interestingly, when OP9 Dll1 co-cultures were initiated with thymic CD34^+^CD1a^+^ progenitors, which are believed to already have committed to the T cell lineage (43), enhanced induction of lineage^−^IL7Rα^+^CRTH2^+^ cells by OP9 Dll1 was not detected (Figure 2C). This indicates that these progenitors might have lost the potential to differentiate into the ILC lineage, as has been demonstrated for murine thymic DN3 cells (23).

### Constitutive NOTCH1 activation robustly induces differentiation of lineage^−^IL7Rα^+^CRTH2^+^ cells

**Figure 3:**
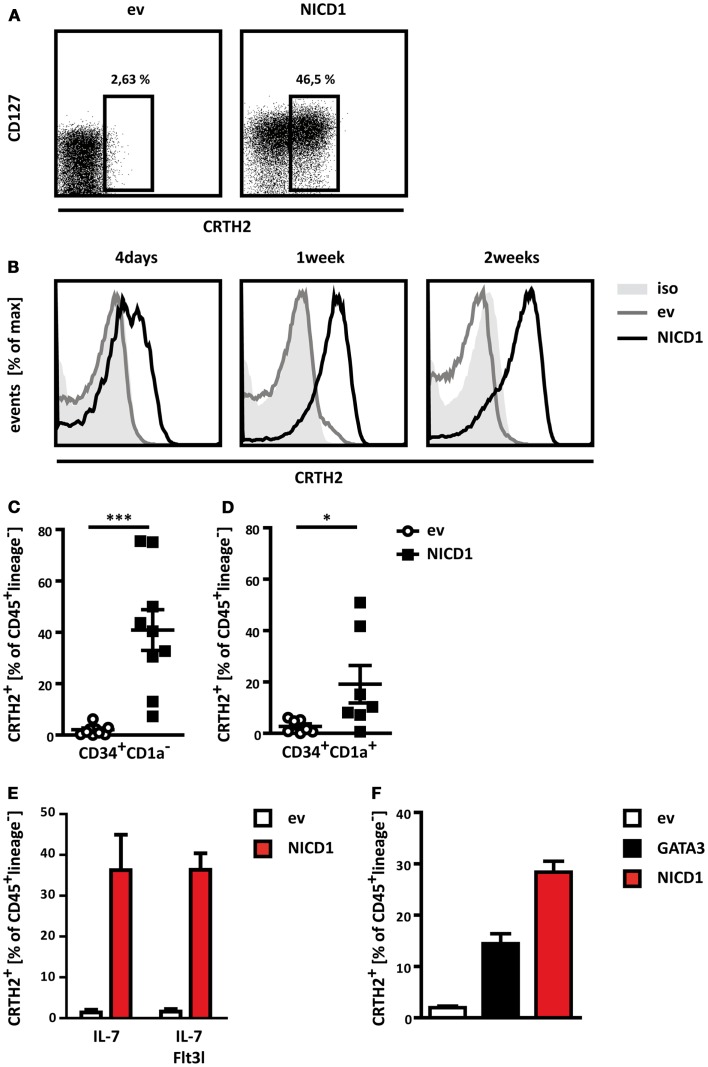
**Constitutive activation of Notch induces prominent ILC2 differentiation**. **(A)** Flow cytometric analysis for expression of CD127 and CRTH2 by thymic CD34^+^CD1a^−^ progenitors transduced with NICD1 (right) or an empty vector control (left), both containing IRES-Thy1.1, and cultured on control OP9 cells. Numbers indicate percentages of CD45^+^lineage^−^ cells expressing CRTH2 among transduced (Thy1.1^+^) cells. Data shown are from one representative experiment out of nine experiments. **(B)** Time course of ILC2 differentiation induced by ectopic expression of NICD1 (black line) in CD34^+^CD1a^−^ thymocytes. CRTH2 expression by transduced CD45^+^lineage^−^ cells after 4 days (left), 1 week (middle), and 2 weeks (right) of co-culture is shown. Data are representative of two independent experiments. Empty vector (ev) control transduced cells are shown in light gray lines, filled dark gray histogram represents staining with an isotype-matched control antibody. **(C,D)** Cumulative data from ILC2 differentiation induced by ectopic expression of NICD1 in CD34^+^CD1a^−^ **(C)** and CD34^+^CD1a^+^ **(D)** thymocytes. Shown is mean ± SEM of nine **(C)** and seven **(D)** experiments. Significance was analyzed using the Mann–Whitney test, *p < 0.05, ***p < 0.0005. **(E)** Differentiation of CD45^+^lineage^−^CRTH2^+^ cells by CD34^+^CD1a^−^ thymocytes cultured in the presence of IL-7 only or IL-7 + Flt3l. **(F)** Differentiation of CD45^+^lineage^−^CRTH2^+^ cells by ectopic expression of NICD1 or GATA3 in CD34^+^CD1a^−^ thymocytes. Data in **(E,F)** are shown as mean + SD from two independent experiments.

OP9 cells expressing Dll1 have widely been used to induce Notch dependent T cell differentiation (44). It is not immediately obvious how activation of Notch in the same progenitors would induce two distinct differentiation programs, namely ILC2 or T cells. Although Notch is clearly required in a cell intrinsic manner in thymic progenitors during T cell differentiation (45), this has not been established for ILC2. Indeed, it is conceivable that ILC2 induction by OP9 Dll1 would be an indirect effect from lateral Notch activation in the OP9 cells, resulting in production of other signals by these cells, which promote ILC2 differentiation. To test whether cell intrinsic Notch signaling in thymic progenitors induces ILC2 differentiation, we ectopically expressed the intracellular domain of NOTCH1 (NICD1) in thymic CD34^+^CD1a^−^ progenitors, thereby inducing constitutive activation of NOTCH1 in these cells, and subjected these to co-culture on control OP9 cells. NICD1 expression resulted in robust induction of an IL7Rα^+^CRTH2^+^ population, which lacks expression of T cell (CD1a, CD3, CD4, CD8, TCRαβ, TCRγδ) and other lineage markers (CD11c, CD14, CD19, CD34, CD94, CD123, FcϵR1, BDCA2) (Figure 3A). Thus, direct activation of Notch in thymic CD34^+^CD1a^−^ progenitors results in the differentiation of cells resembling ILC2 cells.

A prominent population of lineage^−^CRTH2^+^ cells was usually present after 1 week, but these cells first appeared after as little as 4 days of co-culture on OP9 cells, and persisted for up to 2 weeks (Figure 3B). NICD1 induced differentiation of lineage^−^IL7Rα^+^CRTH2^+^ cells was subject to donor-to-donor variation. However, ectopic NICD1 expression consistently induced a prominent lineage^−^IL7Rα^+^CRTH2^+^ population (Figure 3C).

Strikingly, NICD1 even robustly induced a lineage^−^IL7Rα^+^CRTH2^+^ population from T-committed CD34^+^CD1a^+^ thymic progenitors, although to a lesser degree than from uncommitted CD34^+^CD1a^−^ thymocytes (compare Figures 3C,D). This suggests that strong, sustained Notch signaling can overcome commitment to the T cell lineage in thymic progenitors and therefore challenges the lineage fidelity of these precursors. Strikingly, ectopic expression of NICD1 in CD34^+^CD1a^−^ thymocytes also resulted in a prominent lineage^−^IL7Rα^+^CRTH2^+^ population when thymic progenitors were cultured in the combined presence of IL-7 and Flt3l (Figure 3E), conditions known to promote development into the T cell lineage (46, 47).

Collectively, these data show that activation of Notch in human thymic hematopoietic progenitors activates the ILC2 differentiation program. Indeed, the connection between Notch and the ILC2 differentiation program is potent enough to dismantle the T cell differentiation program in cells already committed to the T cell lineage and make them adopt the ILC2 fate instead.

The transcription factor GATA3 has been shown to play an essential role in development and function of both murine and human ILC2 (32, 33). Overexpression of GATA3 in a population of lineage^−^CD127^+^CD117^+^Nkp44^−^CRTH2^−^ immature ILC was sufficient to drive these cells into the ILC2 lineage (33). To determine whether GATA3 is also able to elicit ILC2 differentiation from thymic progenitors, we ectopically expressed this factor from a retroviral vector in CD34^+^CD1a^−^ uncommitted progenitors and monitored ILC2 differentiation. Indeed, GATA3 expression was sufficient to induce an ILC2 phenotype (Figure 3F), further supporting the notion that thymic progenitors have the capacity to differentiate into genuine ILCs. However, NICD1 was more potent in ILC2 differentiation in direct comparison with GATA3, at least at the expression levels obtained by our retroviral expression systems (Figure 3F).

### NOTCH1 induced CRTH2^+^ cells are *bona fide* ILC2

Thus far, our interpretation that Notch activation instructs thymic progenitors to differentiate into ILC2 cells has been based on the absence of lineage markers (including those defining T cells) and the presence of CRTH2, a marker supposedly not found in immature T cells. However, given the wealth of data demonstrating the role of Notch in differentiation of T cells, it is conceivable that the cells obtained here represent a previously not described subtype of immature T cells. Also, it is possible that our results reflect atypical expression of CRTH2 on T cell lineage cells, for instance as a consequence of direct transactivation of the gene encoding this marker by Notch. We therefore sought to determine whether the CRTH2^+^ cells differentiated from thymic progenitors in response to Notch are genuine group 2 ILCs.

**Figure 4:**
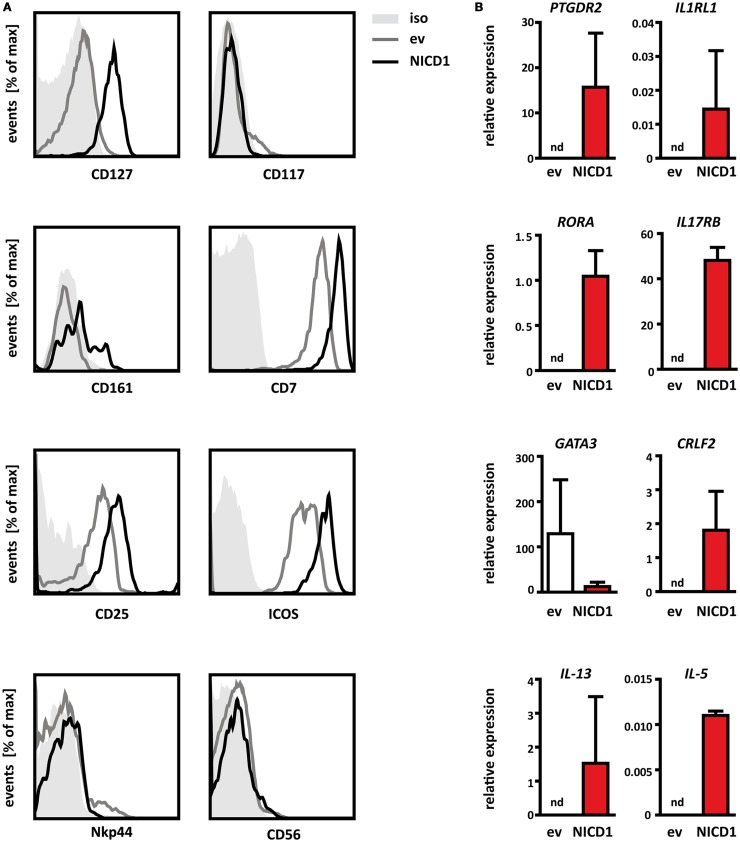
**NICD1 induced lineage^−^CRTH2^+^ cells are *bona fide* ILC2**. **(A)** Flow cytometric analysis of surface expression of CD127 (IL7Rα), CD117 (cKit), CD161, CD7, CD25, ICOS, CD56, and Nkp44 on Thy1.1^+^ thymocytes transduced with NICD1-IRES-Thy1.1-MSCV (black line) or IRES-Thy1.1-MSCV empty vector (ev, gray line) and differentiated *in vitro* on control OP9 cells. Gray shaded: isotype-matched control antibody. FACS data are representative of minimally three experiments. **(B)** Expression of *PTGDR2* (CRTH2), *IL1RL1* (ST2), *IL17RB* (IL-25R), *CRLF2* (TSLP-R), *RORA, GATA3, IL-5*, and *IL-13* mRNA in thymocytes transduced and differentiated *in vitro* as in **(A)**. Cells were sorted after 1 week of culture as NICD1 (red bars) or empty vector transduced (ev, white bars) on the basis of Thy1.1 expression. Expression levels were normalized to the housekeeping gene β-Actin. nd, Not detectable. Data are shown as mean + SD of two independent experiments.

To this end, we performed more extensive phenotyping by multi-color flow cytometry, examining the surface expression of several markers known to be associated with human ILC2. As described above (Figure 3A), NICD1^+^ (CRTH2^+^) cells express the IL7Rα chain (Figure 4A), although surface levels varied between experiments and were sometimes lower than those found on freshly isolated ILCs. This is most likely due to *in vitro* culture in the presence of recombinant IL-7, which results in receptor internalization (48). Indeed, such down regulation of IL7Rα has also been observed when freshly isolated mature ILC2 cells were cultured *in vitro* (9). NICD1 induced CRTH2^+^ cells express low levels of KLRB1 (CD161) in a bimodal distribution (Figure 4A) as has been shown for human ILC derived from other tissues (9). Additionally, NICD1 induced CRTH2^+^ cells also express CD25 (the IL-2Rα chain), CD7, and ICOS, all of which have been shown to be expressed by group 2 ILCs (1, 9). CRTH2^+^ cells differentiated *in vitro* by Notch activation for the most part do not express cKit (Figure 4A). The interpretation of this observation is complicated by the fact that expression of cKit by human group 2 ILC is variable. Among human fetal gut and peripheral blood ILC2, both a cKit^+^ and cKit^−^ population have been described (9). Expression of cKit, therefore, does not seem to constitute part of the core ILC2 identity. Finally, NOTCH1 induced CRTH2^+^ cells do not show surface expression of CD56 and Nkp44 (Figure 4A), associated with the NK and other ILC subsets (1). Taken together, the expression pattern of these surface markers shows that CRTH2^+^ cells derived from thymic progenitors by Notch mediated *in vitro* differentiation phenotypically resemble *bona fide* ILC2 found in the thymus and various other human tissues.

To further characterize these Notch induced thymic ILC2, we determined mRNA expression levels of lineage defining transcription factors, cytokine receptors, and signature cytokines in cells directly isolated from differentiation cultures (Figure 4B). Cells differentiated from thymic progenitors *in vitro* by expression of NICD1 expressed transcripts for the lineage specific cytokine receptors *IL1RL1* (ST2, a subunit of the IL-33 receptor), *IL17RB* (a subunit of the receptor for IL-25), and *CRLF2* (TSLP-R). Together with CD25 (Figure 4A), these cells thus express the critical receptors to respond to the ILC2 activating cytokines IL-2, IL-25, IL-33, and TSLP. Notably, expression of transcripts for these cytokine receptors was not detected in empty vector control cells (Figure 4B). *In vitro* differentiated ILC2 also expressed *RORA* mRNA encoding the ILC2 lineage specific transcription factor RORα. However, these cells did not express elevated levels of *GATA3* compared to control cells. This may seem counter-intuitive, given the ability of GATA3 to induce ILC2 differentiation from thymic progenitors (Figure 3E) and the fact that GATA3 is a known direct target of Notch signaling (49–51). However, it should be noted that expression of GATA3 is low also in resting ILC2 derived from peripheral tissues after *in vitro* culture and that expression of this factor is elevated only after exposure to activating stimuli such as TSLP (33). Therefore, high constitutive expression of GATA3 does not seem to be a characteristic of resting human ILC2 cells. Most strikingly, even without exogenous activation, NICD1^+^ cells expressed transcripts for the ILC2 signature cytokines *IL-13* and *IL-5* (Figure 4B), whereas empty vector control cells did not, further reinforcing the interpretation that NICD1 induced CRTH2^+^ cells are fully functional group 2 ILC. Constitutive expression of IL-5 and IL-13 transcripts by unstimulated ILC2 has been observed previously in the fetal gut (9). We therefore conclude that ILC2 differentiated *in vitro* from thymic progenitors by activation of NOTCH1 resemble *bona fide* group 2 ILCs.

### Notch promotes T cell versus ILC2 differentiation in a signal strength dependent manner

The fact that activation of Notch can induce differentiation of both T cells and ILC2 from the same thymic progenitors raises the question what determines which of these differentiation programs is turned on. The Notch pathway is sensitive to signal amplitude, which allows induction of discretely different responses by one and the same signaling pathway (52, 53). Given our findings that expression of NICD1 was much more potent at eliciting ILC2 differentiation than co-culture with OP9 Dll1, we reasoned that the strength of the Notch signal might be a critical factor deciding whether T cells or ILC2 are generated.

**Figure 5:**
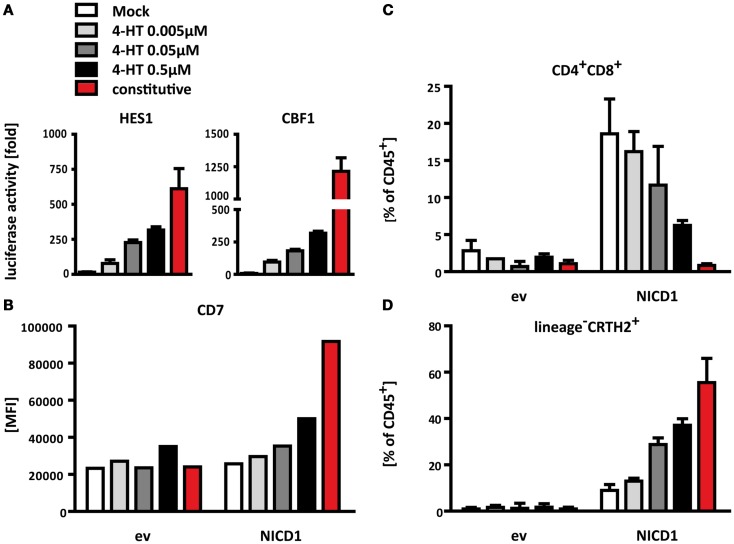
**Notch promotes differentiation of T cells versus ILC2 in a signal strength dependent manner**. **(A)** Activation of the Notch-responsive reporter constructs HES1-luciferase (left) and CBF-1-luciferase (right) induced by different levels of nuclear release of mER-NICD1 or constitutive NICD1 expression. U2OS cells were transfected with a mixture of reporter plasmids expressing Firefly luciferase, a plasmid constitutively expressing Renilla luciferase and an empty vector control, mER-NICD or NICD1, respectively. 4-Hydroxytamoxifen (Tamoxifen, 4-HT) was added at the indicated concentrations. Firefly luciferase activities were normalized to Renilla luciferase activities from the same samples and are displayed as fold of empty vector control samples at the respective concentration of Tamoxifen. Data shown are from one representative of three independent experiments performed in triplicate (mean + SD). **(B–D)** Flow cytometric analysis of thymocytes after 2 weeks of co-culture on control OP9 cells. CD34^+^CD1a^−^ progenitors were transduced with NICD1, mER-NICD1, or an empty vector control prior to co-culture. Tamoxifen was added to mER-NICD1 and empty vector transduced cultures at the concentrations indicated. **(B)** Levels of CD7 expression are displayed as MFI. Data shown are from one representative experiment from 2 similar experiments. **(C)** Transduced cells were analyzed for surface expression of CD4 and CD8 to assess T cell differentiation. **(D)** ILC2 differentiation as determined by expression of CRTH2 on transduced lineage^−^ cells. Data displayed in **(C)** and **(D)** are cumulative from three independent experiments (mean + SD).

To test this hypothesis, we generated an expression construct, in which the concentration of nuclear NICD1 can be controlled in a quantitative manner. In this construct, NICD1 is N-terminally fused to a mutated Estrogen receptor ligand binding domain (mER). This mutated domain no longer binds Estrogen, but does respond to Tamoxifen (54). In the absence of this drug, mER-NICD1 is bound by heat shock proteins in the cytoplasm and hence kept transcriptionally inactive. Addition of Tamoxifen to the culture medium induces transcriptional activity of mER-NICD1 in a dose-dependent manner (Figure 5A). Some leakiness is frequently observed with these types of ER-fusion proteins (55, 56) and indeed, we also consistently observed weak but significant activity of mER-NICD1 in the absence of Tamoxifen (Mock) on two different Notch-responsive promoters (Figure 5A). Therefore, this tool enabled us to induce different levels of NOTCH1 activation, and even explore the impact of very low levels owing to the leakiness of the mER system. In comparison, expression of constitutively active NICD1 served to induce high signaling strength. Together, these constructs allowed us to examine the consequences of Notch signaling across a more than 100-fold dynamic range, as measured in reporter gene assays (compare luciferase activities in the absence of Tamoxifen with those obtained with expression of constitutively active NICD1 in Figure 5A).

To test whether Notch signal strength affects ILC2/T cell differentiation, we retrovirally expressed mER-NICD1 or constitutive NICD1 in uncommitted, CD34^+^CD1a^−^ thymic precursors, subjected these to co-culture on control OP9 cells and titrated in Tamoxifen. The activity of the mER-NICD1 and NICD1 constructs could also be controlled quantitatively in these cells, as shown by the Tamoxifen dose-dependent induction of CD7 expression (Figure 5B), which we have found to be a sensitive gauge for Notch activity in thymic precursors.

Using this system, we measured differentiation of T cells as well as group 2 ILC by flow cytometry. We chose to examine the development of these cells after 2 weeks because of the different kinetics in T cell and ILC2 differentiation. Differentiation of T cells generally requires incubation periods of at least 2 weeks (57) (Figure 2B). As shown before (Figure 3B), group 2 ILC populations emerge within several days of culture, but persist longer and could therefore be assessed here after 2 weeks in direct comparison with T cells.

As expected, neither T cell differentiation nor generation of ILC2 occurred effectively in the absence of Notch activation: considerable populations of both CD4^+^CD8α^+^ T cells (Figure 5C) and lineage^−^IL7Rα^+^CRTH2^+^ ILC2 (Figure 5D) only arose from cultures which had received a Notch signal by means of ectopic NICD1 expression, but not in empty vector transduced control cultures. With regard to dosage dependence, however, T cell and ILC2 differentiation displayed opposite requirements: even very low doses of Notch activity were sufficient to elicit development of T-lineage cells (Figure 5C). In fact, the lowest levels of NOTCH1 signaling activity, owing to the leakiness of our system in the absence of Tamoxifen (see above), gave rise to the most prominent T cell differentiation (almost 20% of CD4^+^CD8α^+^ T cells), whereas hardly any ILC2 were observed in this condition (Figure 5D). Titrating in Tamoxifen to induce higher levels of NOTCH1 signaling gradually diminished the number of T cell lineage cells obtained (Figure 5C). In contrast, stronger NOTCH1 induced more ILC2 differentiation (Figure 5D). Most strikingly, the highest level of Notch signaling, induced here by constitutively active NICD1, did not yield any T cells (Figure 5C); instead, constitutive NICD1 expression resulted in the most prominent ILC2 differentiation (Figure 5D). Taken together, these results demonstrate that by varying signal strength, one and the same Notch pathway can activate two distinct differentiation programs, with weaker signals favoring development of T cells and stronger signals inducing more efficient ILC2 differentiation.

## Discussion

ILC are increasingly recognized as important mediators of immunity and lymphoid tissue (re)modeling (2). The mechanisms underlying differentiation of these cells and the anatomical locations where these processes take place are only beginning to be characterized. What is known has mostly been learned from studies in mice. Here, we have studied the differentiation of human ILC2.

We report the identification of ILC2 in the human thymus. The presence of ILC2 in the thymus could theoretically be explained by migration to this site, although it is not clear what the function of such recruitment would be. The currently known function of ILC2 consists of defense at epithelial barriers (58) and it seems unlikely that ILC2 would be required for this purpose in a sterile internal organ such as the thymus. It is of course conceivable that ILC2 have additional, not identified functions, for instance in tissue homeostasis or development, which explain their presence in this organ. An alternative explanation for their presence in the thymus is that ILC2 are in fact generated in this organ, although the thymus is likely not the only site of ILC2 development. At face value, a role for the thymus in differentiation of ILC2 may seem at odds with a study reporting the identification of a Lin^−^Sca1^hi^Id2^hi^ GATA3^hi^ (LSIG) ILC2 precursor in murine bone marrow (20, 32). However, transcriptome analysis and adoptive transfer experiments suggested that these cells have already committed to the ILC2 lineage and may represent an immature ILC2 stage. Whether differentiation of progenitors into this immature ILC2 stage could also take place in the thymus was not addressed directly in these studies. Another mouse study directly addressed the question whether the thymus is essential for ILC2 development (23). Wong and colleagues made use of FoxN1^nu/nu^ (nude) mice, which display defective thymus development (59). Normal numbers of group 2 ILC were found in mesenteric lymph nodes in these mice after injection of IL-25 (23). While this formally proves that ILC2 differentiation can occur at extrathymic sites, at least in mice, this study does not address whether this process also occurs in the thymus.

Whether ILC2 differentiation occurs in the human thymic organoid could be investigated using humanized immune system mice (60). Furthermore, studies of patients with DiGeorge syndrome, who have defects in thymic development like FoxN1^nu/nu^ (nude) mice (61), could provide valuable insights. However, it stands to reason that, if ILC2 do develop in the thymus, progenitors with the capacity to differentiate into group 2 ILC must exist in this organ. Our finding that CD34^+^CD1a^−^ cells differentiate into ILC2 in response to activation of Notch shows that this is indeed the case.

Recent studies underline the close genetic relationship between T and ILC2 cells, the latter expressing a multitude of markers which classically have been considered T-lineage genes, such as CD7, Lck, Lat, Bcl11b, and Tcf7 (6, 35). Indeed, thymic lineage^−^CD34^+^CD1a^−^cells can develop into both ILC2 and T cells, as we show here. Expression of CD1a by these cells has been considered to mark their commitment to the T cell lineage (43). Correspondingly, we found that CD34^+^CD1a^+^ progenitors failed to give rise to ILC2 after co-culture on OP9 Dll1. However, the ability to differentiate into ILC2 was not lost completely by these cells, as ectopic expression of the NICD1, which we found to be a much stronger stimulus for ILC2 differentiation than OP9 Dll1 cells, could still divert these cells into ILC2. A gradual decrease in ILC2 potential was reported also for murine progenitors: ILC2 differentiation *in vitro* was induced efficiently by OP9 Dll1 when CD44^+^CD25^−^CD4^−^CD8^−^double negative 1 (DN1) cells were used, less efficiently when using CD44^+^CD25^+^ DN2 cells, and not at all with CD44^−^CD25^+^ DN3 cells (23), which are thought to be fully committed to the T cell lineage (62).

Many studies have shown that thymic CD34^+^CD1a^−^ cells differentiate into T cells when cultured on OP9 stroma cells expressing the Notch ligand Dll1 (46, 63). It was surprising, therefore, that differentiation of ILC2 was also obtained using the same conditions and progenitors in our experiments. Our data reveal that the strength of the Notch signal is an important parameter in this decision, with lower Notch signals mediating T cell differentiation, while strong Notch signals induce ILC2 differentiation. Similar dosage dependent outcomes of Notch signaling have been reported before (64), for instance in the lineage decision between αβ and γδ T cells (52, 65). While we did not observe γδ T cells in our cultures, possibly due to differences in culture conditions and progenitor sort strategies, the block in αβ T cell development observed here with high levels of Notch activation is in line with these studies (65). Two non-mutually exclusive mechanisms can be envisioned to explain how a subset of progenitor cells receives the strong Notch signals required for differentiation of ILC2. First, specialized niches with high levels of Notch ligands may exist in the thymus and/or hematopoietic organs (66, 67). Both cortical and medullary epithelial cells (TEC) express the Notch ligands Dll1, Dll4, Jag1 and Jag2 in humans (66). However, other components of the thymic stroma, for instance thymic DCs (68), also express Notch ligands. Second, the CD34^+^CD1a^−^ population may contain a mixture of cells with high and low sensitivity to Notch ligands. Such differential sensitivity may for instance be achieved through modification by Fringe glycosylases, expression of different levels of Notch and downstream mediators or pathway modifiers (69, 70). This diversification may itself be influenced by factors produced in specialized niches. Indeed, factors such as TGFβ, Wnt, and type I interferons can all modify the maximum amplitude of Notch signaling through various ways (64) (Amsen, unpublished data). TECs produce TGFβ (71), while thymic plasmacytoid DCs constitutively express IFNα under non-inflamed conditions (72), indicating once more that both epithelial and non-epithelial stromal components of the thymic microenvironment might contribute to specialized niches capable of eliciting strong Notch signals in developing thymocytes. Given that mice lacking thymic epithelial cells still generate ILC2 (23), it is tempting to speculate that the relevant source of the Notch activating signals consists of a non-epithelial stromal cell.

The prominent induction of ILC2 differentiation by strong Notch signaling echoes a similar role for Notch in differentiation of T helper 2 cells, which are functionally related to ILC2 (73). Induction of Th2 differentiation by Notch involves direct transactivation of GATA3 in CD4^+^ T cells (31, 50, 51). Since GATA3 is also essential for the generation and function of murine and human ILC2 (32, 33), it seemed likely that Notch mediated ILC2 differentiation would also proceed via induction of this transcription factor, at least partially. To our surprise, however, GATA3 levels were not elevated by Notch signaling during differentiation of ILC2 cells from CD34^+^CD1a^−^ cells. Although not elevated, GATA3 expression was still clearly detectable in these cells, suggesting that Notch mediated ILC2 development might not be completely independent of GATA3. Nonetheless, it seems likely that Notch induces the ILC2 fate predominantly via activation of other genes. Interestingly, some of the genes shared between the T cell and ILC2 programs (6, 35), Tcf1 and Bcl11b, are established direct targets of Notch (74, 75). Direct transactivation of such genes would provide at least a partial explanation for the ability of Notch to induce ILC2 differentiation without elevating expression of GATA3, although it seems likely that also other direct Notch targets exist, which are dedicated to the ILC2 differentiation program. One mechanistic explanation for the high Notch signal strength dependence of ILC2 differentiation might be that transactivation of such dedicated target genes requires higher concentrations of NICD, for instance due to steric impediment by factors surrounding the Notch-responsive elements.

In summary, we show that the human thymus contains ILC2. Whether or not this reflects a role for this organ in development of these cells, progenitors with the capacity to differentiate into ILC2 can be found in the thymus. These progenitors reside within a population which also has the capacity to generate T cells in response to apparently the same set of signals and we demonstrate that the strength of Notch signaling is an important determinant in deciding which fate is chosen. Apart from augmenting our understanding of the processes involved in the generation of ILC2, these findings also suggest possible avenues to generate ILC2 for reconstitution of this cell type in patients after stem cell transplantation or in patients suffering from immunodeficiencies.

## Materials and Methods

### Isolation of thymic hematopoietic progenitors

Postnatal thymic (PNT) tissue specimens were obtained from children undergoing open heart surgery (LUMC, Leiden, The Netherlands); their use was approved by the AMC ethical committee in accordance with the declaration of Helsinki. Cell suspensions were prepared by mechanical disruption using the Stomacher 80 Biomaster (Seward). After overnight incubation at 4°C, thymocytes were isolated from a Ficoll-Hypaque (Lymphoprep; Nycomed Pharma) density gradient. Single cell suspensions were enriched for CD34^+^ cells by MACS (Miltenyi Biotec), stained with fluorescently labeled antibodies and subsequently FACS sorted on a FACS Aria (BD Bioscience) as CD34^+^CD1a^−^CD3^−^CD56^−^BDCA2^−^ or CD34^+^CD1a^+^CD3^−^CD56^−^BDCA2^−^, respectively (referred to in this study as CD34^+^CD1a^−^ and CD34^+^CD1a^+^). Purity of the sorted populations was >99%.

### Flow cytometry

Staining for expression of surface proteins was performed at 4°C for 20 min. Distinction of live and dead cells was based on staining with 7-Aminoactinomycin D (7-AAD, eBiosciences) or fixable live/dead dyes (Invitrogen). Data were acquired on a LSR Fortessa flow cytometer (BD Bioscience) and analyzed using FlowJo software (TreeStar). Single cell suspensions were stained with antibodies directly labeled with Fluorescein Isothiocyanate (FITC), Phycoerythrin (PE), Phycoerythrin-Cyanine 5 (PE-Cy5), PE-Cy5.5, PE-Cy7, PerCP-Cy5.5, Allophycocyanin (APC)/Alexa Fluor 647, APC-Cy7, AF700 (all BD Bioscience, BioLegend, or MACS Miltenyi), Horizon V500 (HV500, BD Bioscience), Brilliant Violet 421 (BV421), BV711, and BV785 (all BioLegend). Antibodies specific for the following human antigens were used: CD1a, CD3, CD4, CD7, CD8, CD11c, CD14, CD19, CD25, CD34, CD45, CD56, CD94, CD117 (cKit), CD123, CD127 (IL7Rα), CD161, CD294 (CRTH2), CD303 (BDCA2), CD336 (Nkp44), CD278 (ICOS), TCRαβ, TCRγδ, and FcεR1. Anti-mouse CD90.1 (Thy1.1) -FITC, -PE, or -APC-eFluor 780 (eBioscience) were used to detect cells transduced with MSCV – IRES-Thy1.1 retroviruses.

### Retroviral constructs

The human NICD1-IRES-Thy1.1-MSCV construct has been described before (49). To generate the mER-NICD fusion, an N-terminal mER domain was PCR amplified using the following primers: GATCAGGAATTCCACACCATGGGAGATCCACG AAATGAA and GATCAGGATATCCACCTTCCTCTTCTTCTTGG and cloned into the EcOR1 and EcORV sites of pBluescript (pBS) to create mER-pBS. Human NICD1 lacking a translation initiation signal was PCR amplified using these primers: ATCGGAGGTTCTCGCAAGCGCCGGCGGCAGCAT and GATCAGAAGCTTGAATTCTTACTTGAAGGCCTCCGGAATG and subsequently cloned into the EcORV and *Hind*III sites of mER-pBS. The mER-NICD1 fusion insert was then cloned into IRES-Thy1.1-MSCV using BamH1 and Cla1.

### Virus production and transduction

For virus production, Phoenix GALV packaging cells were transiently transfected using FuGene HD (Promega). Virus containing supernatant was harvested 48 h after transfection, snap frozen on dry ice and stored at −80°C until use. For transduction, cells were incubated with virus supernatant in plates coated with Retronectin (30 μg/ml, Takara Biomedicals) for 6–8 h at 37°C the following day.

### *In vitro* differentiation of thymic progenitors

Sorted thymic progenitors were cultured overnight in Yssel’s medium containing 5% normal human serum, SCF (20 ng/ml), and IL-7 (10 ng/ml, both PeproTech). OP9 cells were mitotically inactivated by irradiation with 30 Grey and seeded at a density of 5 × 10^3^/cm^2^ 1 day prior to initiation of co-cultures. After transduction, thymic progenitors were added to pre-seeded OP9 cells. Co-cultures were performed in MEMα (Invitrogen) with FCS (20%, FetalClone I, Hyclone) and IL-7 (5 ng/ml). In some cases, Flt3l (5 ng/ml, PeproTech) was added to the medium. Cultures were refreshed every 3–4 days. Differentiation assays for ILCs were typically analyzed after 1 week, unless stated otherwise. Cells were harvested by forceful pipetting and passed through 70 μm nylon mesh filters (Spectrum Labs). For longer culture periods, cells were transferred onto fresh stromal cells every week.

### Quantitative real-time PCR

RNA was isolated using the NucleoSpin RNA II or NucleoSpin RNA XS kit (Macherey-Nagel) according to the manufacturer’s instructions. cDNA synthesis was done using the High Capacity cDNA kit (Applied Biosystems). Quantitative Real-Time PCRs were performed in an iCycler instrument using IQ SYBR Green Supermix (both BioRad). For calculation of relative expression level of the genes of interest, the ΔCt method was used. The following primers were used: *ACTB* (β-*Actin*) (Gene ID: 60) Forward: CAC CAT TGG CAA TGA GCG GTT C; Reverse: AGG TCT TTG CGG ATG TCC ACG T; *CRLF2* (*TSLP-R*) (Gene ID: 64109) Forward: GAG TGG CAG TCC AAA CAG GAA; Reverse: ACA TCC TCC ATA GCC TTC ACC; *GATA3* (Gene ID: 2625) Forward: ACC ACA ACC ACA CTC TGG AGG A; Reverse: TCG GTT TCT GGT CTG GAT GCC T; *IL1RL1* (*ST2*) (Gene ID: 9173) Forward: ATG TTC TGG ATT GAG GCC AC; Reverse: GAC TAC ATC TTC TCC AGG TAG CAT; *IL-5* (Gene ID: 3567) Forward: AGC TGC CTA CGT GTA TGC CA; Reverse: CAG GAA CAG GAA TCC TCA GA; *IL-13* (Gene ID: 3596) Forward: ATT GCT CTC ACT TGC CTT GG; Reverse: GTC AGG TTG ATG CTC CAT ACC; *IL17RB* (Gene ID: 55540) Forward: CCA ACA CAG CAC TAT CAT CG; Reverse: ATA TGG AGT CAG CTG CAC CG; *PTGDR2* (*CRTH2*) (Gene ID: 11251) Forward: AAT CCT GTG CTC CCT CTG TG; Reverse: ATG TAG CGG ATG CTG GTG TT; *RORA* (Gene ID: 6095) Forward: ACA AGC AGC GGG AGG TGA TGT; Reverse: TGA GAG TCA AAG GCA CGG C.

### Reporter gene assays

U2OS cells were transiently transfected using the FuGene HD transfection reagent (Promega). Cells were co-transfected with a NOTCH-responsive promoter and either NICD1-IRES-Thy1.1-MSCV, mER-NICD1-Thy1.1-MSCV, or an empty vector control. To correct for differences in transfection efficiency, the pRL-CMV control vector was co-transfected, from which Renilla luciferase is expressed constitutively. Transfections were performed in triplicate. Where applicable, 4-Hydroxy-Tamoxifen (Sigma) was added after overnight incubation to induce nuclear translocation of mER-NICD1. Cells were lysed 48 h post transfection and luciferase activity was measured using the Dual Luciferase Reporter Assay System (Promega) on a Synergy HT microplate reader (Syntek). Two different Notch-responsive reporter constructs were used, which have been described previously (76).

## Authors Contribution

Rebecca Gentek designed, performed, and analyzed experiments and wrote the manuscript. J. Marius Munneke and Christina Helbig performed and analyzed experiments. Bianca Blom, Mette D. Hazenberg, and Hergen Spits designed and analyzed experiments. Derk Amsen designed and analyzed experiments and wrote the manuscript.

## Conflict of Interest Statement

The authors declare that the research was conducted in the absence of any commercial or financial relationships that could be construed as a potential conflict of interest.
